# Fabrication of Surfactant-Free Mixed-Metal Nanocatalyst–Carbon Fiber Paper Composites via Pulsed Laser Grafting

**DOI:** 10.1021/acs.jpcc.5c00641

**Published:** 2025-04-24

**Authors:** Madeleine K. Wilsey, Teona Taseska, Lydia R. Schultz, Elena Perez, Astrid M. Müller

**Affiliations:** † Materials Science Program, University of Rochester, Rochester, New York 14627, United States; ‡ Department of Chemical Engineering, University of Rochester, Rochester, New York 14627, United States; § Department of Chemistry, University of Rochester, Rochester, New York 14627, United States

## Abstract

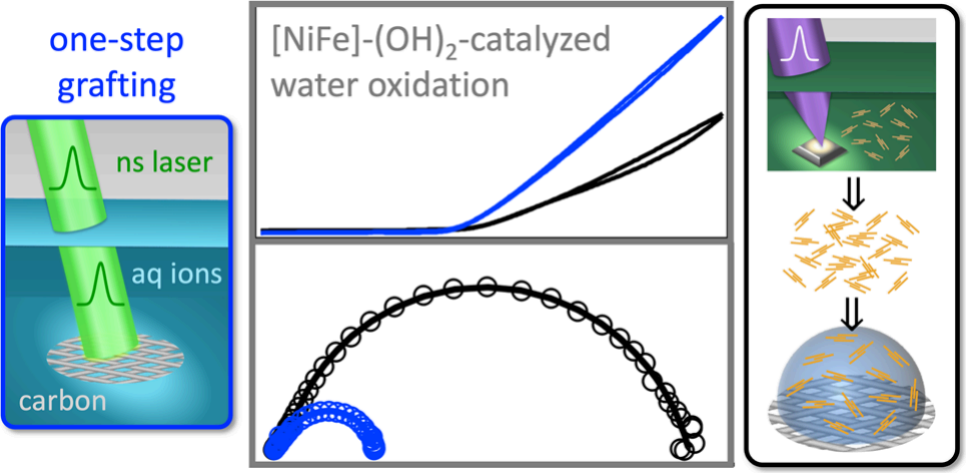

We present a novel methodology for fabricating surfactant-free mixed-metal nanocatalyst–carbon fiber paper composites, demonstrating significant improvements in impedance, electrocatalytic activity, and long-term stability over laser synthesized drop cast analogues on carbon fiber paper or highly ordered pyrolytic graphite. Our innovative pulsed laser grafting technique is a versatile, one-step aqueous process that integrates nanoparticle generation with surface attachment on macroscopic solid supports, such as sheets, rather than being limited to powders, particulate supports, or organic solvents as in prior methods. It effectively addresses longstanding challenges with nanoparticle adhesion and electrical contact between nanoparticles and macroscopic electrodes, and it alleviates environmental concerns associated with organic solvents. Laser grafting eliminates laborious synthesis, separation, purification, and postsynthesis attachment steps, thus significantly reducing composite preparation time. We fabricated [NiFe]-(OH)_2_–hydrophilic carbon fiber paper composites using aqueous nickel–iron nitrate solution. Low-fluence 532 nm nanosecond laser pulses minimized surface damage and facilitated effective metal ion excitation for nanoparticle assembly. SEM, EDX and XPS data revealed surface [NiFe]-(OH)_2_ without carbon encapsulation and prominent Ni–C interactions. The pulsed laser grafted composites showed enhanced electrocatalytic performance for alkaline water oxidation and decreased material charge transfer resistance, compared to drop cast analogues, leading to improved electrical conductivity and mass activity. Additionally, they demonstrated exceptional long-term stability, overcoming common adhesion issues in conventional nanoparticle–support systems, marking a significant advancement in the manufacturing of multimetallic nanoparticle–support composites, with promising implications for electrochemistry and electrocatalysis technologies.

## 1 Introduction

The development of viable electrocatalysis technologies is essential for global decarbonization and the remediation of environmental pollutants, highlighting the need for efficient and durable nonprecious electrodes.^1−3^ The effectiveness of electrocatalytic technologies critically depends on electrode performance, which can be enhanced by using nanoparticles on high surface area conductive supports.^1,4−6^ Designing rapid, customizable, scalable, and reproducible processes to manufacture efficient and durable nanoparticle-containing electrodes is key for advancing electrocatalysis innovations. Water oxidation plays a critical role in decarbonization technologies, as it generates the protons and electrons necessary for fuel-forming transformations from abundant small-molecule feedstocks.^5,7^ Additionally, water oxidation can be utilized in aqueous advanced oxidation processes to remediate environmental pollutants, including per- and polyfluoroalkyl substances (PFAS).^2,8−11^ For global scalability, the materials employed in water oxidation must be nonprecious.^5^

Nickel–iron layered double hydroxide, which contains solely earth-abundant elements, is among the most efficient and stable catalysts for alkaline water oxidation.^5,12,13^ Layered double hydroxides are mineral-like materials with the general formula [M^II^_1–*x*_ (M′)^III^_*x*_(OH)_2_](A^*m*–^)_(*x*/*m*)_·*n*H_2_O, where M^II^ represents divalent metals and (M′)^III^ denotes trivalent metals, while A^*m–*^ signifies an anion. Their most stable composition range is 0.2 ≤ *x* ≤ 0.4. Layered double hydroxides consist of sheets of edge-shared M^II^(OH)_6_ octahedra, in which varying amounts of (M′)^III^ atoms occupy some of the M^II^ sites. Hydroxide ligands extend into the interlayer galleries, which also contain water molecules. Intercalated anions serve to balance the excess positive charge resulting from the substitution of M^II^ by (M′)^III^.^14^ The chemical identity, charge and amount of the intercalated anions, as well as the amount of intergallery water control the basal plane spacing and the electrical and ionic conductivity of layered double hydroxides.^15−21^ Nanocatalysts often outperform bulk materials because of the high surface area-to-volume ratio and enhanced reactivity at the nanoscale due to a higher number of undercoordinated surface sites and quantum effects.^1,5,22,23^ Nanoparticles can be prepared with precisely controlled properties, such as size, shape, and composition,^24−26^ as demonstrated with nickel–iron layered double hydroxide nanosheets.^5,12,27^

Utilization of nanoparticles in electrochemistry applications necessitates supports to provide electrical contact to the power source. Carbon is a premier electrode support material because of its high overpotential for many electrocatalytic reactions.^28−30^ Carbon fiber paper is particularly useful because of high surface area, affordability, and scalability, making it a common carbon electrode support material for electrochemical transformations.^31^ In aqueous applications, like water oxidation, hydrophilicity is essential to effectively utilize the large internal surface areas of porous carbon supports.^32^ Despite extensive research on nanosized electrocatalysts,^33−41^ nanoparticles are not broadly used in industrial electrolyzer systems because of widespread issues with nanocatalyst adhesion and electrical contact.^42−45^ Weak adhesion is detrimental for the stability of nanoparticle–support composites, and poor electrical contact leads to high impedance, lowering energy efficiency. These challenges make the attachment of nanoparticles on electrode supports the premier obstacle in the manufacturing of nanocatalyst-containing electrodes.

The importance of strong interactions between nanocatalyst and catalyst support electrode materials in electrolyzers has been emphasized in the literature.^46^ The mobility of nanoparticles during electrocatalysis can lead to nanoparticle growth^47^ and nanocatalyst agglomeration, which negatively impacts the electrical conductance of the integrated electrode,^48,49^ as higher catalyst dispersion has been correlated with enhanced catalytic activity.^50^ Nanoparticle–support interactions have been found to be stable in certain widely used industrial processes, such as platinum group metals on metal oxide supports (e.g., ceria and alumina) for diesel exhaust gas treatment,^51−53^ and nanoparticles on activated carbon powders for wastewater treatment via ultrasonication,^54,55^ although the latter is notably energy-intensive.^2^ While these are important catalytic processes for environmental remediation, decarbonization efforts require electrolyzers capable of storing carbon-neutral electricity in the chemical bonds of e-fuels, making electrocatalytic processes in electrolyzers essential.^3^ For such processes, freestanding electrodes are necessary, requiring the attachment of nanocatalysts to conductive electrode support materials.^48^ Although Pt/C catalysts are commonly used in current industrial fuel cell devices, they continue to face inherent stability challenges,^56−60^ which has led to the use of polymer binders to enhance catalyst adhesion.^61,62^

Nanoparticle attachment to supports is usually accomplished on the laboratory scale through electrostatic methods or by using ion-conducting polymer (ionomer) binders,^63,64^ such as Nafion,^65^ either as overlayers or mixed with the catalyst nanoparticles as inks.^66−68^ However, electrostatic methods lack long-term stability,^69−71^ and Nafion can corrode non-acid-stable catalysts or carbon supports,^5,72,73^ reducing electrode stability. Alkaline electrolytes can mitigate acid-related issues but negatively affect the ion conductivity of Nafion.^65,74^ Furthermore, binders have been shown to increase the ohmic resistance in proton exchange membrane electrolyzer cells used for hydrogen production, thereby limiting overall efficiency.^75,76^ Additionally, the use of binders complicates reaction mechanisms because ionomers change the catalyst microenvironment.^77,78^ Likewise, surfactants, which conventional nanoparticle syntheses require for size and shape control,^79,80^ alter the interface between the catalyst surface and the electrolyte, with the added complication of the limited stability of nanoparticle–surfactant bonds during electrochemical reactions.^81−83^

Overall, binders and surfactants impede close interaction between nanoparticles and their supports, resulting in poor electrical contact and energy efficiency in composite electrodes. Nanoparticles produced by pulsed laser in liquid synthesis are free of surfactants.^1^ However, like all nanoparticulate catalysts—regardless of synthesis method—laser-synthesized nanoparticles must be attached to a freestanding conductive support to function as electrodes,^48,84^ and face similar challenges related to adhesion, durability, electrical contact, and energy efficiency as discussed above. While pulsed laser in liquid synthesis has made significant strides in generating colloids and depositing them onto powder supports,^85−87^ the effective attachment of nanoparticles to electrically conductive, freestanding supports—essential for electrical contact with the nanocatalysts—remains an unresolved challenge. Although the industrial synthesis of supported nanoparticles has been well established for powder-based or nonconductive metal oxide supports,^88,89^ translating this to freestanding conductive electrode supports, as opposed to particulate or powder substrates, continues to be a major challenge.^53^ Moreover, even for powder supports, the spatial distribution of nanoparticles is a critical factor influencing catalytic performance.^90^ Additionally, the uniform postsynthetic attachment of catalyst nanoparticles to freestanding electrochemical supports is difficult to scale to large electrode areas,^91^ can lead to nanoparticle aggregation,^92^ which is associated with electrode failure,^93^ and often lacks reproducibility.^94−97^

Self-supported electrocatalysts can be fabricated using various single- or multistep strategies that allow control over composition, size distribution, elemental dispersion, surface morphology, and catalytic activity.^91^ Multistep approaches, which are inherently complex and labor-intensive, typically involve either the separate synthesis of nanocatalysts followed by their deposition onto a freestanding conductive support, or the deposition of precursors onto substrates with subsequent composition-specific post-treatment.^91^ Existing one-step methods involve the in situ growth of catalysts directly on conductive substrates using techniques such as electrochemical deposition, electrophoresis, hydro/solvothermal synthesis, or wet chemical/sintering processes.^91^ Atomic layer deposition has also been employed for this purpose.^98^ While these methods have enabled the preparation of nanoelectrocatalysts on conductive supports at the laboratory scale, they have inherent limitations in terms of scalability, particularly with respect to uniformity over large electrode areas and manufacturing throughput. For instance, electrochemical deposition is constrained by the achievable nanomaterial dimensions and morphologies, and maintaining consistency over large areas remains difficult due to the sensitivity of the process to reaction conditions.^99,100^ Electrophoretic deposition suffers from poor morphology control, weak film adhesion, and declining deposition rates over time.^101,102^ Hydro/solvothermal methods often face challenges such as uneven heating, prolonged reaction times, phase separation, and polydispersity, in addition to requiring autoclaves that pose safety concerns for scale-up.^103,104^ Wet chemical and sintering routes demand precise reaction control, complicating uniformity and reproducibility at larger scales, and typically require surfactants that can interfere with catalytic performance.^105^ Atomic layer deposition requires vacuum and is constrained by its processing time, with typical deposition rates of 100–300 nm h^–1^ for the best systems.^106,107^ In contrast, the pulsed laser grafting method of this work offers a versatile and inherently scalable alternative, as it operates in aqueous media under ambient conditions. It is particularly well-suited for large-area manufacturing at scale due to the widespread industrial adoption of laser-based processing and scanning technologies.^108−112^

Laser reduction in liquid has been reported as an effective method for producing metal particles on support materials. For example, the laser deposition of gold with an approximate transverse size of 1 μm from Au^I^–triphenylphosphine complexes in chloroform onto glass, Ge, Si, SiO_2_, or GaAs substrates was first reported in 1986.^113^ Additionally, pulsed laser irradiation of molten transition metal salts has been used to deposit gold and copper metals with lateral dimensions of approximately 1 μm onto Si and GaAs semiconductors in barrier contact with the substrate.^114^ More recently, high-entropy alloy nanoparticles have been synthesized via laser scanning ablation of precursor-impregnated carbon materials, including particulate supports such as carbon nanofibers and graphene, as well as carbonized wood, all laser processed under hexane to exclude oxygen.^115^ These approaches are challenging to scale over large areas due to the requirement for anaerobic conditions, the use of high-temperature molten salts, and environmental concerns associated with organic solvents. A more scalable and sustainable strategy would employ techniques that operate under ambient conditions in aqueous media.

Here, we report the development of a novel strategy employing pulsed laser processing in liquid to directly graft mixed-metal nanocatalysts onto hydrophilic carbon fiber paper from dissolved transition metal precursors in an aqueous solution. A key innovation of this pulsed laser grafting approach is its compatibility with freestanding, macroscopic solid supports such as carbon fiber paper, thereby overcoming the limitations associated with powder or particulate supports. Carbon fiber paper is widely used in electrochemical applications—including fuel cells, electrolyzers, supercapacitors, lithium-ion batteries, sensors, water desalination, and wastewater treatment—as well as in biosensing, tissue engineering, regenerative medicine, and cancer research.^116−138^ The capability to graft mixed-metal nanoparticles directly from aqueous solution onto a macroscopic carbon support represents a transformative step forward in the scalable fabrication of surfactant-free and binder-free nanoparticle–freestanding support composites, and distinguishes our method from previously reported laser-based deposition techniques, which are largely limited to nano- and micromaterials on particulate supports.^1^ Pulsed laser grafting significantly advances laser-based material deposition beyond particulate supports by enabling effective grafting onto a stationary, macroscopic substrate. Notably, heat dissipation from a static macroscopic carbon fiber paper sheet is inherently less efficient than from particulate matter dispersed and moving within a liquid, where the larger surface area per material mass and increased convective effects enhance thermal transport.^139^ The successful grafting of nanocatalysts under these less favorable heat transfer conditions highlights the distinctive and impactful nature of the pulsed laser grafting process.

The pulsed laser grafting methodology effectively addresses the longstanding challenges associated with postsynthesis attachment by directly seeding and growing nanoparticles on the carbon fiber paper support in a single step, using nanosecond laser pulses. This approach eliminates the laborious separate synthesis, separation, purification, and nanoparticle attachment steps that are required by other synthesis methods, thus significantly accelerating the fabrication of integrated composites. Our study reveals the interaction between laser parameters and the optical properties of both the grafting liquid and the solid support. Pulsed laser grafted [NiFe]-(OH)_2_ on hydrophilic carbon fiber paper exhibited superior performance for alkaline water oxidation, compared to drop cast laser synthesized [NiFe]-(OH)_2_ nanocatalysts, showing increased catalytic activity, reduced impedance, and enhanced durability.

## 2 Experimental Methods

All chemicals were used as received. Deionized water was obtained from a Thermo Scientific Barnstead Smart2Pure Pro UV/UF 15 LPH Water Purification System and had a resistivity of ≥ 17.5 MΩ · cm. All experiments were conducted at room temperature and in ambient air. The glassware was subjected to cleaning with aqua regia, followed by extensive rinsing with distilled water and drying. Data analysis and graphing were performed with Igor Pro 8.04 (Wavemetrics), unless otherwise stated.

### 2.1 Composite Preparation

Hydrophilic carbon fiber paper was prepared as support for the [NiFe]-(OH)_2_ catalyst. Specific details of this preparation are detailed elsewhere.^32^ To summarize, as purchased carbon fiber paper (FuelCell Store, AvCarb MGL190) was sonicated for 5 min in 1.0 M aqueous sodium dodecyl sulfate solution (AG Scientific, ≥99%) followed by electrooxidation at +1.63 V vs Ag/AgCl for 20 min in 0.1 M aqueous KHCO_3_ solution (pH 8.7, Alfa Aesar, 99.7%–100.5%). Hydrophilic carbon fiber paper was thoroughly rinsed and dried with nitrogen from liquid nitrogen boil-off.

Laser-grafted [NiFe]-(OH)_2_ on hydrophilic carbon fiber paper composites were prepared by placing a 1.5 cm (wide) × 2.5 cm (long) piece of hydrophilic carbon fiber paper, with the top right corner cut off to indicate the face-up side of the carbon fiber paper piece, on a glass flange in a 30 mL beaker and submerging this carbon fiber paper piece in an aqueous solution that contained 0.92 M Ni(NO_3_)_2_ (Alfa Aesar, 98.0%) and 0.08 M Fe(NO_3_)_3_ (Thermo Fisher, 98.0–101.0%), resulting in a Ni/Fe ratio of 12/1 and referred to as 1.0 M aqueous (Ni_12_Fe_1_) nitrate solution. The carbon fiber paper piece was 5 mm below the liquid surface. Photographs of the setup are shown in Figure S1. A magnetic stir bar continuously stirred the solution at 750 rpm. An unfocused 532 nm, 8 ns pulsed laser beam (10 Hz Nd:YAG laser, Spectra-Physics Quanta-Ray LAB-190) with a pulse energy of 680 mW, corresponding to a fluence of 87 mJ cm^–2^, was directed from above into the solution and carbon fiber paper. After 60 min of irradiation, the laser-grafted [NiFe]-(OH)_2_ on hydrophilic carbon fiber paper composite was removed, rinsed with water, and dried in ambient air.

Laser synthesized [NiFe]-(OH)_2_ nanoparticles were prepared using pulsed laser in liquid synthesis, following a protocol reported elsewhere.^12^ Briefly, a 10 Hz Q-switched Nd:YAG laser (Spectra-Physics Quanta-Ray LAB-190), using 90 mJ, 8 ns pulses at 355 nm, was focused with a 10 cm focal length plano-concave fused silica lens 0.5 mm below the liquid surface of a 10 mL 3.0 M aqueous nickel nitrate solution with 0.5 g iron powder (Alfa Aesar, −200 mesh, ≥99%) in 30 mL beaker for 60 min. The solution was stirred at 750 rpm. Following laser synthesis, a strong magnet was used to separate any unreacted iron powder from the suspension of [NiFe]-(OH)_2_ nanoparticles. Using centrifugation, the nanoparticulate power was isolated, washed with water five times, followed by two washes with acetone (VWR), and dried under vacuum. Composites were prepared by drop casting aqueous suspensions of 0.04 mg mL^–1^ on hydrophilic carbon fiber paper electrodes with a geometric area of 0.09 cm^2^ or highly ordered pyrolytic graphite (HOPG, SPI Supplies) electrodes with a geometric area of 0.09 cm^2^, and dried at 60 °C with a heat lamp for 15 min.

### 2.2 Physical Characterization

Scanning electron microscopy (SEM) images were obtained at UR-Nano. A Zeiss Auriga scanning electron microscope with a Schottky field emission emitter was operated at 20.00 kV, with a working distance of 5.1 mm. Energy-dispersive X-ray (EDX) spectroscopy data were collected using an SEM-integrated EDAX Octane Elect Plus spectrometer with a with silicon drift detector. Double sided carbon tape was used to adhere samples to stubs. Elemental ratios were derived from Gaussian peak fits of background-subtracted EDX spectra, in keeping with accepted protocol.^140^

X-ray photoelectron spectra (XPS) data were collected at UR-Nano using a Kratos Axis Ultra XPS instrument with a monochromatized Al Kα source. The instrument operated at 200 W and 15 kV under a base pressure of 3.0 × 10^–8^ mbar. Dry samples were placed on the sample bar using double-sided adhesive copper tape. Survey scans were acquired from 0 to 1200 eV with 1 eV step size, 200 ms dwell time, and 160 eV pass energy and averaged over 5 scans. High-resolution core level scans were collected with a 0.1 eV step size, 260 ms dwell time, and 20 eV pass energy and averaged over 5 scans. All spectra were calibrated using the C 1s peak at 284.8 eV.^141^ The data were processed in CasaXPS (Version 2.3.24), using Shirley background subtraction,^142^ Gaussian/Lorentzian peak fitting, and instrument-specific atomic sensitivity factors.

X-ray diffraction (XRD) measurements of laser grafted [NiFe]-(OH)_2_ on hydrophilic carbon fiber paper composites were conducted at the Chemical Analysis Lab at the Rochester Institute of Technology. A Bruker D8 ADVANCE diffractometer with Cu Kα radiation (40 kV, 40 mA), a 0.6 mm primary slit, a 5.0 mm secondary slit, a 2.5 mm antiscatter screen, and a Lynxeye detector were used. Each measurement was performed with a resolution of 0.020° in 2θ and 0.5 s per step dwell time, resulting in approximately 40 min per sample. Background subtraction was conducted using Bruker DIFFRAC.SUITE software. XRD data of the laser synthesized [NiFe]-(OH)_2_ nanoparticles were collected using a Rigaku XtaLAB Synergy-S diffraction system equipped with a HyPix-6000HE HPC detector. A light coating of viscous oil affixed the powder samples to a Nylon loop (0.1 mm ID). A PhotonJet-S microfocus source at 50 kV generated 1 mA CuKα radiation (λ = 1.54184 Å). Two combination ω–φ “Gandolfi” scans were performed, each for 300 s: (1) ω from −62.00° to 31.00° and φ rotated through 720°, at θ = −42.127 and κ = 70.000°; (2) ω from −31.00 to 61.00 degrees and φ rotated through 720°, at θ = 40.877 and κ = −70.00°. A sample-to-detector distance of 34 mm was used.

Optical spectra were obtained using a fiber-optic ultraviolet to near-infrared optimized spectrometer (OCEAN-HDX-XR). The same 1 cm or 1 mm path length quartz cuvettes were used to obtain the spectra of the three solutions, to exclude any manufacturing inconsistencies between cuvettes. Air blanks were used, and spectra of water were collected and subtracted from spectra of aqueous solutions of 0.92 M Ni(NO_3_)_2_, 0.08 M Fe(NO_3_)_3_, or 1.00 M (Ni_12_Fe_1_) nitrate.

### 2.3 Electrochemical Data

Potentiostatic Electrical Impedance Spectroscopy (PEIS) data were collected at an applied potential of 1.7 V vs the reversible hydrogen electrode (RHE). The sinusoidal perturbation for PEIS was set to an amplitude of 10 mV, with a frequency range spanning from 100 kHz to 100 mHz. The resolution was set to 10 points per decade with each point being an average of three measurements. The PEIS data were analyzed using the Bio-Logic EC-Lab software package.

Electrocatalytic water oxidation was performed in a standard three electrode single-compartment setup, in keeping with the literature.^5,12,27,47^ Composite anodes consisting of laser synthesized [NiFe]-(OH)_2_ drop cast on HOPG, laser synthesized [NiFe]-(OH)_2_ drop cast on hydrophilic carbon fiber paper, or pulsed laser grafted composites of [NiFe]-(OH)_2_ on hydrophilic carbon fiber paper were used as working electrodes, with geometric mass loadings of 8.8, 8.8, and 8.3 μg cm^–2^, respectively. The counter electrode was nickel mesh (Aldrich, 0.025 mm thick, 99.9%), and a reversible hydrogen reference electrode (Gaskatel Hydroflex) was used. The electrolyte was 1.0 M aqueous KOH with a pH value of 14.0, determined by a Mettler Toledo SevenExcellence pH/Ion/C/DO meter S975-K with a InLab Expert Pro-ISM pH probe. The electrolyte was stirred at 500 rpm. Chronoamperometry data were collected at 2.0 V vs RHE for 30 h. Cyclic voltammetry data were collected from 0.97 to 2.27 V vs RHE with a scan rate of 100 mV s^–1^, in keeping with published data for alkaline water oxidation by laser synthesized nanocatalysts.^27,47,143^

## 3 Results and Discussion

### 3.1 Fabrication of Pulsed Laser Grafted Nanoparticle–Support Composites

**Figure 1:**
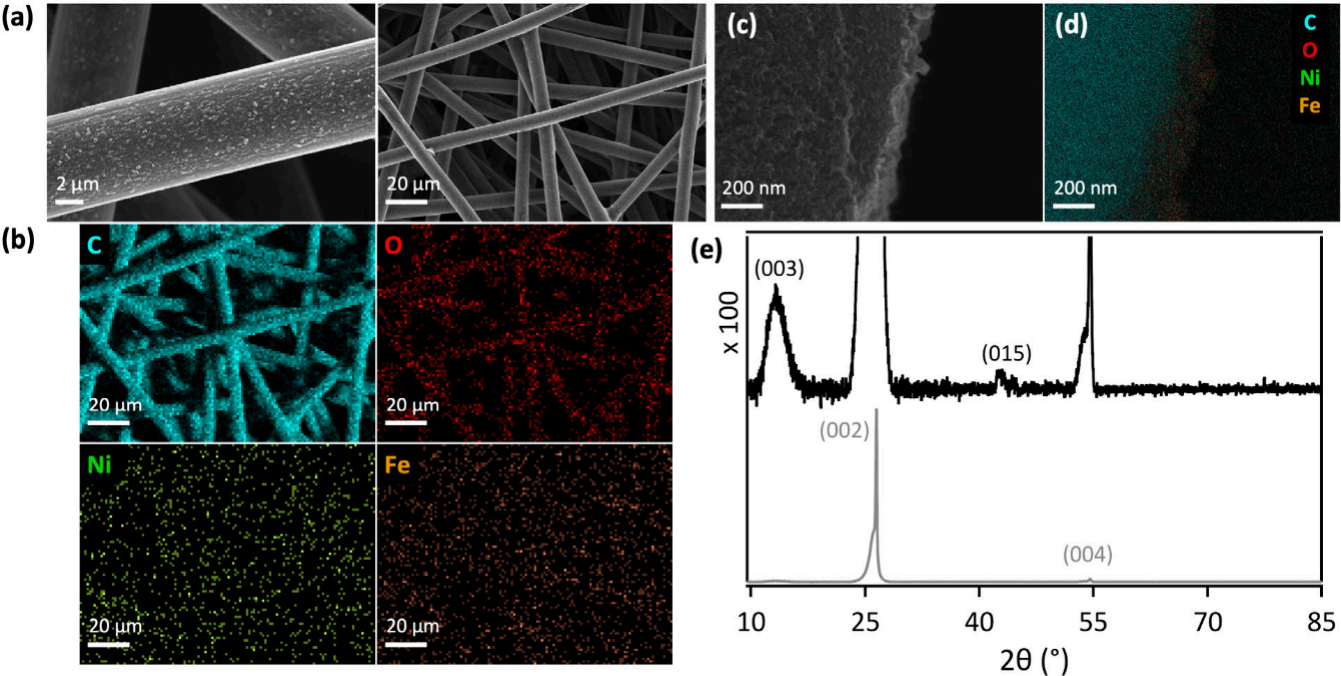
(a) SEM images of pulsed laser grafted [NiFe]-(OH)_2_ on hydrophilic carbon fiber paper composites with (b) EDX maps showing carbon, oxygen, nickel, and iron. (c) Cross-sectional SEM image of pulsed laser grafted [NiFe]-(OH)_2_ on hydrophilic carbon fiber paper with (d) EDX map overlaying carbon, oxygen, nickel, and iron. (e) XRD data of [NiFe]-(OH)_2_ on hydrophilic carbon fiber paper composites where the *y*-axis of the top spectrum (black) was magnified by a factor of 100 to visualize the [NiFe]-(OH)_2_ peaks.

We used pulsed laser grafting to prepare composites of [NiFe]-(OH)_2_ on a high surface area carbon support (Figure 1a), using hydrophilic carbon fiber paper that was immersed in an aqueous nickel and iron nitrate solution. We chose transition metal nitrates because of their high solubility and because pulsed laser in liquid synthesis of [NiFe]-(OH)_2_ nanosheets from iron powder in aqueous nickel nitrate solution has been reported.^1,12,27,143^ The utilization of the composite in the aqueous application water oxidation electrocatalysis and use of an aqueous laser grafting liquid necessitate that the carbon support is hydrophilic. Hydrophilicity enhances water contact with the carbon fiber paper surface,^32^ facilitating interaction with aqueous metal ions^144^ and improving heat transfer.^145,146^ In previous work, we developed an eco-friendly, rapid, and scalable method for rendering carbon fiber paper hydrophilic through graphitic edge carbon oxygenation.^32^ This process creates a high density of graphitic edges on the surfaces of carbon fibers while preserving the integrity of the carbon fiber network, unlike other hydrophilicity-imparting methods that compromise it,^32^ as demonstrated by scanning electron microscopy (SEM) imaging (Figure 1a, right). Pulsed laser grafting resulted in a uniform coverage of the obtained [NiFe]-(OH)_2_ material on hydrophilic carbon fiber paper supports (Figure 1), with a Ni/Fe ratio of 75:25, derived from EDX data (Figure S2a–c). This Ni/Fe ratio falls well within the range of di- and trivalent metal ratios of layered double hydroxides^17,18^ and matches the reported Ni/Fe ratio of laser-synthesized [NiFe]-(OH)_2_.^12,27^ Only C, O, Fe, and Ni were detected, evident from EDX data (Figure S2a–c) and survey XPS data (Figure S3), which both show the absence of other elements. Cross-sectional SEM and EDX data, collected at the interface between a single carbon fiber and laser-grafted [NiFe]-(OH)_2_, demonstrate that the elements nickel and iron were uniformly distributed throughout the catalyst layer (Figure 1c,d), indicating that [NiFe]-(OH)_2_ formed on the carbon fiber surface without the presence of underlying metal oxide structures. Additionally, minimal carbon was detected within the catalyst layer, ruling out carbon encapsulation of [NiFe]-(OH)_2_.

XRD data provide evidence for the layered double hydroxide structure of the laser-grafted nickel–iron material on hydrophilic carbon fiber paper (Figure 1e). The peaks at 2θ values of 26.5 and 54.5° are attributable to the (002) and (004) reflections of hydrophilic carbon fiber paper, respectively, showing the characteristic peak asymmetry with lower-angle shoulders, which arise from underground sample contributions of porous carbon fiber paper.^32^ The (003) and (015) reflections of [NiFe]-(OH)_2_ are visible. These [NiFe]-(OH)_2_ reflections are weak compared to the stronger carbon peaks due to the low catalyst mass loading, which is desirable for achieving high electrocatalytic performance (see section 3.2). Additionally, the typical probe depths in XRD range from a few micrometers to several hundred micrometers, contingent upon the density of the material,^147^ causing the carbon reflections to dominate the [NiFe]-(OH)_2_ signals. The (006) reflection of [NiFe]-(OH)_2_ at a 2θ value of approximately 25° is obscured by the strong (002) reflection of the carbon fiber paper at 26.5°. The (003) reflection of [NiFe]-(OH)_2_ appeared at a 2θ value of 13.4°, corresponding to a basal plane spacing of 6.6 Å, consistent with hydrotalcite-like structure.^17,18,20^ For comparison, the laser synthesized [NiFe]-(OH)_2_ nanosheets showed a (003) reflection at a 2θ value of 11.0° (Figure S4), corresponding to a basal plane spacing of 8.0 Å, consistent with published data on laser synthesized [NiFe]-(OH)_2_.^12,27^ XRD peaks for laser grafted and laser synthesized [NiFe]-(OH)_2_ were broadened, attributable to small crystallite size and stacking faults including turbostratic disorder in the hydrotalcite-like structure, in line with what has been reported for laser synthesized [NiFe]-(OH)_2_ nanosheets.^27^ The [NiFe]-(OH)_2_ nanosheets were laser synthesized by irradiating metallic iron powder targets in 3.0 M aqueous Ni(NO_3_)_2_ solution with 355 nm nanosecond pulses with 1 × 10^5^ J cm^–2^ fluence, following reported protocols.^8−10,12,27^ Pulsed laser in liquid synthesized [NiFe]-(OH)_2_ nanosheets had a Ni/Fe ratio 75:25, derived from EDX data (Figure S5a).

We obtained the pulsed laser grafted [NiFe]-(OH)_2_–hydrophilic carbon fiber paper composites by irradiating hydrophilic carbon fiber paper submerged in 1.0 M aqueous (Ni_12_Fe_1_) nitrate solution for 60 min, using unfocused Nd:YAG laser irradiation with 8 ns, 532 nm, 87 mJ cm^–2^ pulses. The fabrication time for pulsed laser grafted nanoparticle–support composites of 1 h is significantly shorter than typical separate nanoparticle synthesis, purification, and subsequent support attachment of several hours.^148−151^ The carbon fiber paper was submerged in 5 mm deep grafting liquid, to prevent ion depletion and unwanted heating above the carbon fiber paper, which are general challenges of thin liquid films because of transport limitations.^152^ For ns pulses at a wavelength of 532 nm, graphite exhibits an ablation threshold fluence of 0.7 J cm^–2^,^153^ due to its effective absorption coefficient of 5 μm^–1^,^154^ and a critical melting fluence of 0.13 J cm^–2^.^155^ Consequently, the fluence selected for the pulsed laser grafting process of 87 mJ cm^–2^ was well below the carbon ablation and sublimation thresholds. We employed nanosecond laser pulses to minimize surface damage to graphitic carbon fiber paper. Given the thermal time constant of graphite, heat dissipation on the scale of micrometers occurs on the order of a few ns,^156^ rendering nanosecond pulses particularly suitable for minimizing nonlinear excitation effects, which would be induced by shorter pulses.^157−159^ Subnanosecond laser pulses are additionally more costly, posing scalability challenges for composite manufacturing. The use of nanosecond pulses in pulsed laser grafting provides the added benefit of facilitating surface decontamination and activation while enabling the preparation of surfactant-free nanoparticles via pulsed laser in liquid synthesis, which has extensively been used to prepare tailored mixed metal nanomaterials.^1,160−162^ Multimetallic catalysts often outperform monometallic catalysts.^1^ Our novel grafting approach advances traditional nanosecond pulsed laser cleaning technology^163,164^ by activating the support surface, allowing for immediate nanoparticle seeding and growth in a brief decontaminated state, thus enhancing nanoparticle adhesion and electrical contact to the carbon fiber paper support (cf. section 3.2).

**Figure 2:**
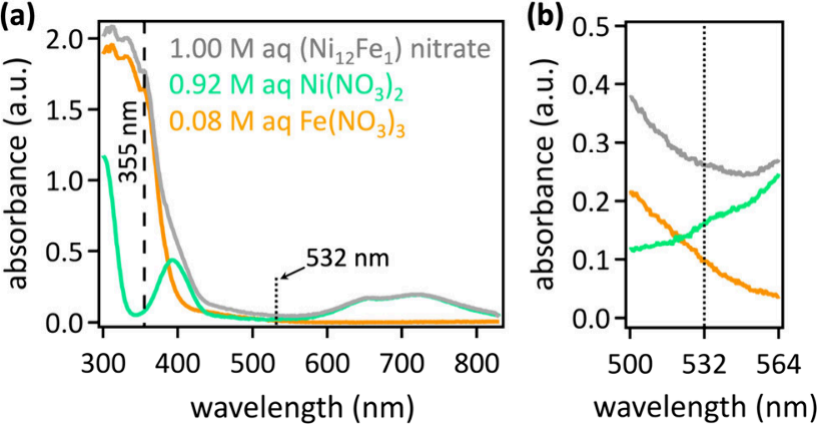
Optical spectra of aqueous (aq) solutions of 1.0 M (Ni_12_Fe_1_) nitrate (gray), 0.92 M Ni(NO_3_)_2_ (green), and 0.08 M Fe(NO_3_)_3_ (orange), collected in a (a) 1 mm or (b) 1 cm optical path length cuvette. The dashed line indicates a wavelength of 355 nm, and the dotted lines mark 532 nm. Note that absorbances above 1.0 suffered from detector saturation and are therefore higher in reality than depicted.

The laser grafting liquid consisted of a 1.0 M aqueous (Ni_12_Fe_1_) nitrate solution, yielding a Ni/Fe ratio of 75:25 in the resulting pulsed laser-grafted nanocatalyst (cf. EDX data above), consistent with the Ni/Fe ratio of well-studied [NiFe]-(OH)_2_ water oxidation catalysts.^5,12,27,165^ We chose a total transition metal ion concentration of 1.0 M because regular pulsed laser in liquid synthesis of [NiFe]-(OH)_2_ nanosheets from iron powder utilized aqueous nickel nitrate solutions with concentrations of 1 to 3 M.^12^ The strong ultraviolet absorption of aqueous ferric nitrate solution^166^ (Figure 2a) precluded use 355 nm pulses for pulsed laser grafting of [NiFe]-(OH)_2_ on hydrophilic carbon fiber paper. Instead, we employed 532 nm laser pulses. Both the nickel and the iron nitrate components of the aqueous grafting liquid exhibit low yet nonnegligible absorbances at 532 nm (Figure 2). The absorbance values for the 5 mm path length that the laser beam traversed are 0.08 and 0.05 for the aqueous Ni(NO_3_)_2_ and Fe(NO_3_)_3_ components, respectively, derived from the data shown in Figure 2b. The growth of [NiFe]-(OH)_2_ nanoparticles from dissolved Ni^2+^ and Fe^3+^ ions during the pulsed laser grafting process requires that the laser light excites both metal ion components, i.e. aqueous Ni(NO_3_)_2_ and Fe(NO_3_)_3_. At the same time, the absorption of the metal ion compounds should be low at the laser wavelength to enable the utilization of a grafting liquid with a high metal ion concentration, such as the one-molar solution used here, to provide enough metal precursors at the solid support–grafting liquid interface for the assembly of nanoparticles. Simultaneously, the apparent absorbance, i.e. that of the aqueous (Ni_12_Fe_1_) nitrate solution, must be sufficiently low at the chosen laser wavelength, as to not attenuate the laser beam too much, to permit nanosecond pulsed laser activation of the solid support. Here, the 532 nm absorbance of the aqueous (Ni_12_Fe_1_) nitrate solution for the 5 mm path length that the laser beam traversed was 0.13, derived from the data shown in Figure 2b. Finally, [NiFe]-(OH)_2_ has an optical spectrum with a broad minimum at 520–600 nm,^167^ minimizing further laser processing of the grafted [NiFe]-(OH)_2_ material during laser irradiation. For all these reasons combined, use of 532 nm laser light enabled the fabrication of pulsed laser grafted composites of [NiFe]-(OH)_2_ on hydrophilic carbon fiber paper.

**Figure 3:**
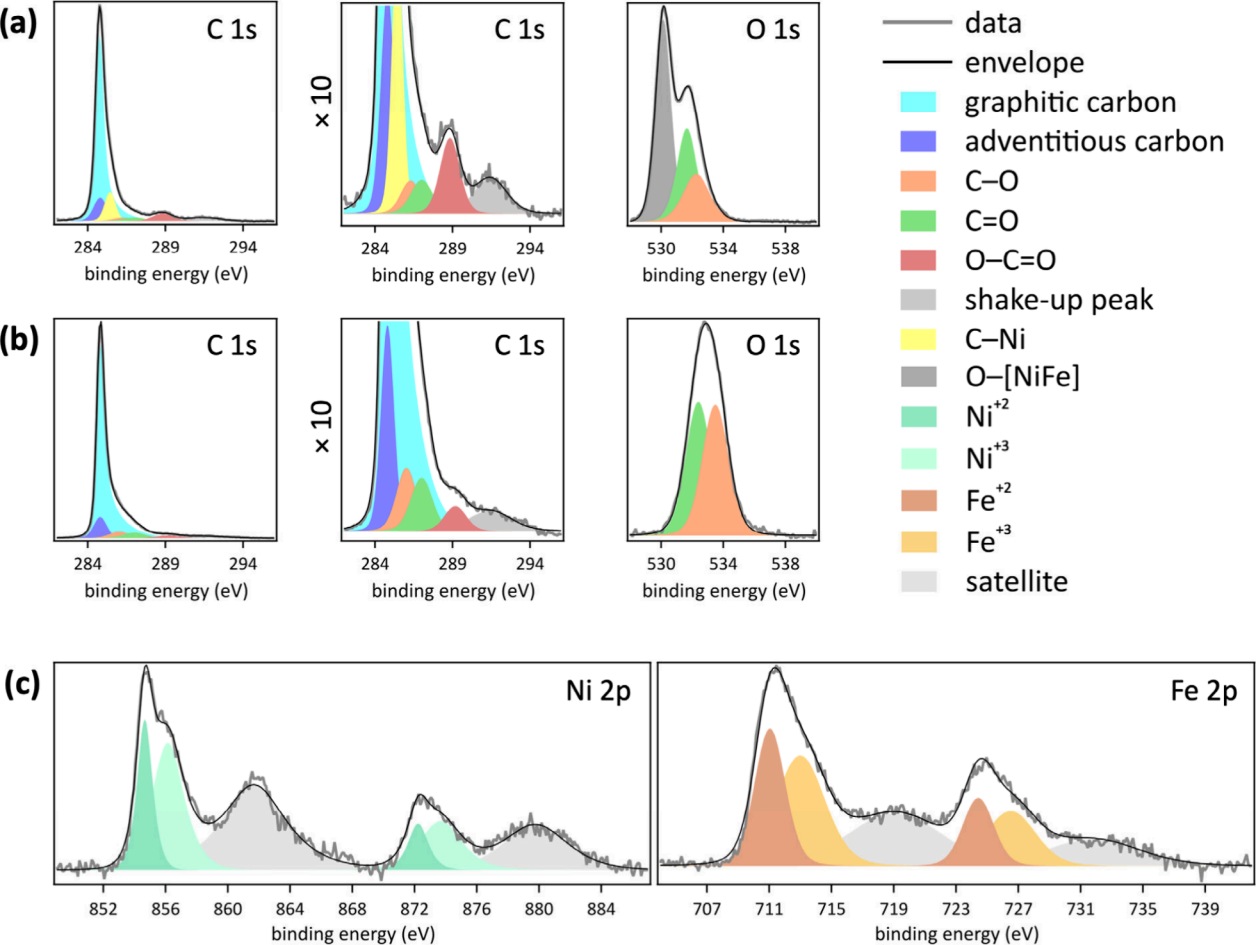
Core level C 1s and O 1s XPS data of (a) pulsed laser grafted [NiFe]-(OH)_2_ on hydrophilic carbon fiber paper composites and (b) neat hydrophilic carbon fiber paper, with center panels with *y*-axes magnified by a factor of 10. (c) Core level Ni 2p and Fe 2p data of pulsed laser grafted [NiFe]-(OH)_2_ on hydrophilic carbon fiber paper composites. Relative contents of XPS species are provided in Table S1.

XPS data provide evidence for the successful fabrication of a composite material by pulsed laser in liquid grafting, in which the [NiFe]-(OH)_2_ catalyst was integrated at the interface of the graphitic hydrophilic carbon fiber paper support (Figure 3). We observed a prominent peak attributable to Ni–C interactions in high-resolution core level C 1s data (yellow peak in Figure 3a). This peak exhibits a central binding energy of 285.4 eV, consistent with a bonding interaction between nickel and carbon,^168^ mimicking the C 1s XPS signature of multilayer benzene on nickel.^169^ This 285.4 eV peak was absent in the core level C 1s region of neat hydrophilic carbon fiber paper, where only carbon and oxygen were detected (Figure 3b), as expected for this material.^32^ The 285.4 eV peak, indicative of Ni–C interactions, was also not observed in the C 1s XPS data of laser-synthesized [NiFe]-(OH)_2_ drop-cast onto otherwise virtually identical hydrophilic carbon fiber paper (Figure S3d). This suggests that pulsed laser grafting promotes a more intimate contact between the [NiFe]-(OH)_2_ catalyst and the carbon fiber paper support than can be achieved by drop-casting a separately synthesized nanocatalyst.

The C 1s core level data of hydrophilic carbon fiber paper required six peaks to fit the measured data, including an asymmetric graphitic carbon peak (284.7 eV), a symmetric adventitious carbon peak (284.8 eV), and broad shakeup peak (291.4 eV), in agreement with reported values.^32,170−173^ The remaining three peaks were assigned to carbon oxygenates, in line with published data,^8,9,32^ with the following central binding energies: C—O at 286.1 ± 0.2 eV, C=O at 287.0 ± 0.2 eV, and O—C=O at 289.0 ± 0.2 eV.^32,172,174,175^ The O 1s core level region of hydrophilic carbon fiber paper was matched by fitting with two peaks with central binding energies of 532.3 and 533.3 eV, corresponding to C=O and C—O bonds in the surface oxygenates, respectively, in keeping with literature values.^32,175,176^ Atom percentages of surface species are in Table S1. We found that the C 1s π–π* shakeup peak increased by a factor of 1.5 for laser-grafted [NiFe]-(OH)_2_ on hydrophilic carbon fiber paper composites, compared to hydrophilic carbon fiber paper, indicative of disorder and contributions from sp^2^ and sp^3^ carbon.^177^ We surmise that this disorder arises from the nanosecond laser treatment, which facilitates the insertion of [NiFe]-(OH)_2_ at the hydrophilic carbon fiber paper surface, corroborated by our observation of prominent Ni–C interactions (Figure 3a). In addition, we detected a 3-fold increase in surface O—C=O species, attributable to interactions between surface oxygenates and the metal hydroxide catalyst. Compared to XPS data of neat hydrophilic carbon fiber paper, the O 1s core level region of laser-grafted [NiFe]-(OH)_2_ on hydrophilic carbon fiber paper composites required an additional peak at 530.1 eV, indicative of the presence of [NiFe]-(OH)_2_ at the surface, consistent with reported central binding energy values for oxygen bonded to metal.^178,179^ Likewise, the surface oxygen content of [NiFe]-(OH)_2_ on hydrophilic carbon fiber paper composites exceeded that of neat hydrophilic carbon fiber paper by a factor of 5. Further, the presence of surface [NiFe]-(OH)_2_ shifted the peaks attributed to C=O and C—O to lower binding energies of 531.7 and 532.3 eV, respectively. Thus, due to the metal oxide catalyst obfuscating the atom percentages of oxygen species, we did not quantify the relative atom percentages of the O 1s components of pulsed laser grafted [NiFe]-(OH)_2_–hydrophilic carbon fiber paper composites.

In addition to surface carbon and oxygen, surface nickel and iron were detected in XPS data of laser-grafted [NiFe]-(OH)_2_ on hydrophilic carbon fiber paper composites (Figure 3c), indicating that nickel and iron were exposed at the surface. This means that unwanted carbon encapsulation of the [NiFe]-(OH)_2_ catalyst did not occur during pulsed laser grafting of [NiFe]-(OH)_2_ on hydrophilic carbon fiber paper. Carbon encapsulation and carbonaceous shells are often observed in pulsed laser in liquid synthesis of metals and metal oxides in carbon-containing media.^1,180^ Carbonaceous overlayers block catalytically active sites, impeding catalytic activity.^1^ High resolution data of the Ni 2p region were matched by fitting with three spin–orbit doublets corresponding to Ni^2+^, Ni^3+^, and satellite peaks. Peaks at 854.7 and 872.2 eV were assigned to Ni^2+^ 2p_3/2_ and Ni^2+^ 2p_1/2_, respectively, while the peaks at 856.1 and 873.7 eV were assigned to Ni^3+^ 2p_3/2_ and Ni^3+^ 2p_1/2_, respectively,^181^ corroborating the presence of surface [NiFe]-(OH)_2_. The corresponding satellite peaks had central binding energy values of 861.7 eV (2p_3/2_) and 879.8 eV (2p_1/2_).^181^ Likewise, the core level Fe 2p region spectrum was matched by fitting with three spin–orbit doublets corresponding to Fe^2+^, Fe^3+^, and satellite peaks. Peaks at 711.0 and 724.4 eV were assigned to Fe^2+^ 2p_3/2_ and Fe^2+^ 2p_1/2_, respectively, while the peaks at 713.0 and 726.5 eV were assigned to Fe^3+^ 2p_3/2_ and Fe^3+^ 2p_1/2_, respectively.^181^ The corresponding satellite peaks had central binding energy values of 719.1 eV (2p_3/2_) and 731.7 eV (2p_1/2_).^181^ Our observation of surface Ni^2+^, Ni^3+^, Fe^2+^, and Fe^3+^ suggests that the [NiFe]-(OH)_2_ material consisted of the typical [Ni^II^Fe^III^]-(OH)_2_ as well as phases that contained Ni^III^ and Fe^II^. The formation of Fe^II^-containing nickel–iron layered hydroxide has been reported.^165^ Nickel–iron hydroxides conform to the general formula of layered double hydroxides of [M^II^_1–*x*_(M′)^III^_*x*_(OH)_2_](A^*m*–^)_(*x*/*m*)_·*n*H_2_O.^14^ Mixed-valent first-row transition metals are commonly found in minerals.^182−184^ Trivalent nickel is known to occur in [Ni^III^Fe^III^]-(oxy)hydroxide. Preparation of such (oxy)hydroxides typically requires anodic potentials, and controlled synthesis of mixed-metal (oxy)hydroxides outside of electrochemical conditions is challenging.^185−187^ Pulsed laser in liquid synthesis is known to enable the formation of nonequilibrium nanomaterials.^1^ Surface Ni^2+^, Ni^3+^, Fe^2+^, and Fe^3+^ species have been observed in reported XPS data of hydrothermally synthesized nickel–iron layered double hydroxides.^188,189^ Under alkaline water oxidation conditions, all nickel–iron hydroxide phases convert into the [Ni^III^Fe^III^]-(oxy)hydroxide,^5,10,190,191^ which is the resting state of the catalyst under turnover.^143^ Therefore, the initial speciation of nickel and iron is irrelevant for employing the [NiFe]-(OH)_2_ on hydrophilic carbon fiber paper composite as the anode in alkaline water oxidation electrocatalysis (cf. section 3.2).

**Figure 4:**
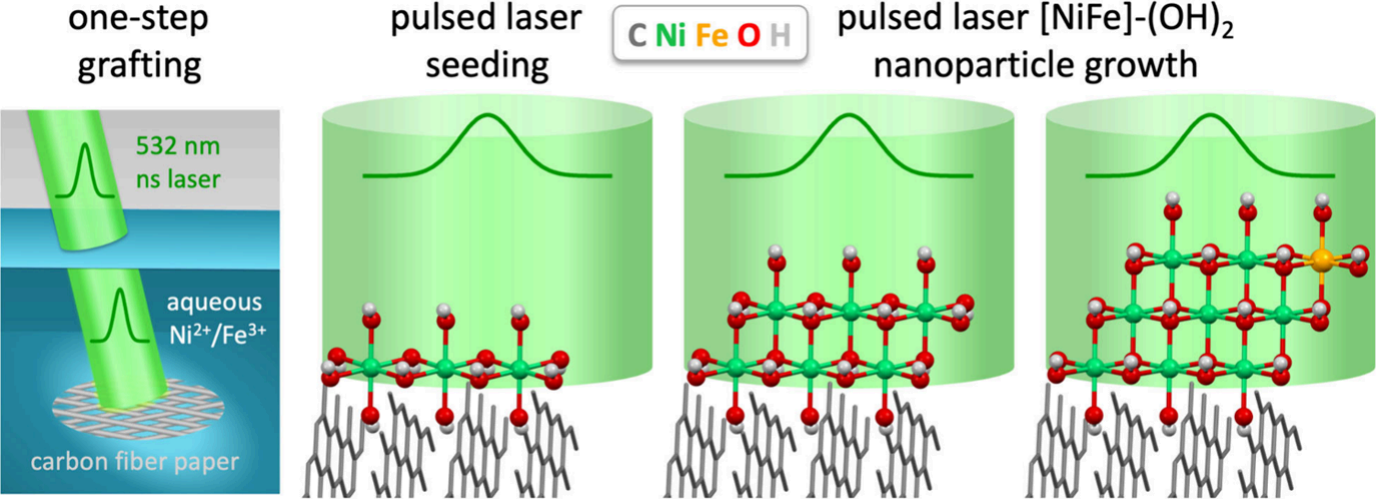
Schematic illustration of the one-step pulsed laser grafting process that combines the nanosecond laser-induced activation/decontamination of the graphitic hydrophilic carbon fiber paper with seeding and growth of [NiFe]-(OH)_2_ catalyst nanoparticles at graphitic edges, leading to [NiFe]-(OH)_2_–hydrophilic carbon fiber paper composites with intimate contact between the catalyst and the support. The oxygenates on the hydrophilic carbon fiber paper are omitted for visual clarity.

Taken together, the SEM, XPS, and optical spectroscopy data show that we succeeded in fabricating [NiFe]-(OH)_2_–hydrophilic carbon fiber paper composites by our newly developed one-step pulsed laser grafting methodology for mixed-metal nanomaterials on carbon surfaces. The data additionally demonstrate that our nanosecond pulsed laser grafting process enables (1) the activation of the hydrophilic carbon fiber paper support, evident from the increased C 1s π–π* shakeup peak; (2) the seeding and embedding of oxidized nickel sites into the carbon surface, presumably at graphitic edges, established by the detection of a prominent C 1s XPS peak due to Ni–C interactions; and (3) the growth of [NiFe]-(OH)_2_ nanoparticles without carbonaceous shells at the carbon support surface, shown by the detectability of surface nickel and iron by XPS, which only detects surface-exposed elements. The schematic in Figure 4 illustrates this proposed mechanism of nanoparticle–support composite fabrication by nanosecond pulsed laser grafting. The process includes the activation of the liquid-immersed solid substrate surface by the nanosecond laser pulse, laser-induced seeding of metal sites from dissolved ions, and laser-assisted growth of nanoparticles at these seed sites, to assemble mixed-metal hydroxide nanoparticles.

### 3.2 Electrochemistry of Pulsed Laser Grafted Nanoparticle–Support Composites

We assessed the electrical contact between laser-made [NiFe]-(OH)_2_ nanocatalysts and carbon supports by potentiostatic electrochemical impedance spectroscopy (PEIS). Electrochemical impedance can be used as a quantitative measure for the electrical contact between nanoparticles and supports.^192−194^ PEIS data are represented in a Nyquist plot, which shows the negative imaginary impedance (Z) versus the real impedance.^28^ In a Nyquist plot, the presence of a resistance (R) and a capacitance (C) in parallel, called an RC loop, is indicated by semicircles, with a larger semicircle radius signifying greater resistance and capacitance, and consequently, poorer electrical contact.^28^ Because our hydrophilic carbon fiber paper electrodes are porous and nonflat, they do not behave as ideal capacitors. Therefore, we used constant phase elements instead of parallel-plate capacitors.^195−197^ In keeping with the vast electrochemistry literature on [NiFe]-(OH)_2_ materials for alkaline water oxidation,^198−202^ we assessed PEIS data, obtained under anodic potential, instead of collecting EIS data at open circuit potential, where no faradaic current flows, which precludes water oxidation turnover. Because [NiFe]-(OH)_2_ is a layered material that contains water and hydroxide anions in the interlayer galleries,^17,20,27,203^ the resistivity of [NiFe]-(OH)_2_ changes during alkaline water oxidation turnover, compared to the catalyst at open circuit potential, thus making PEIS data more meaningful.^204^

**Figure 5:**
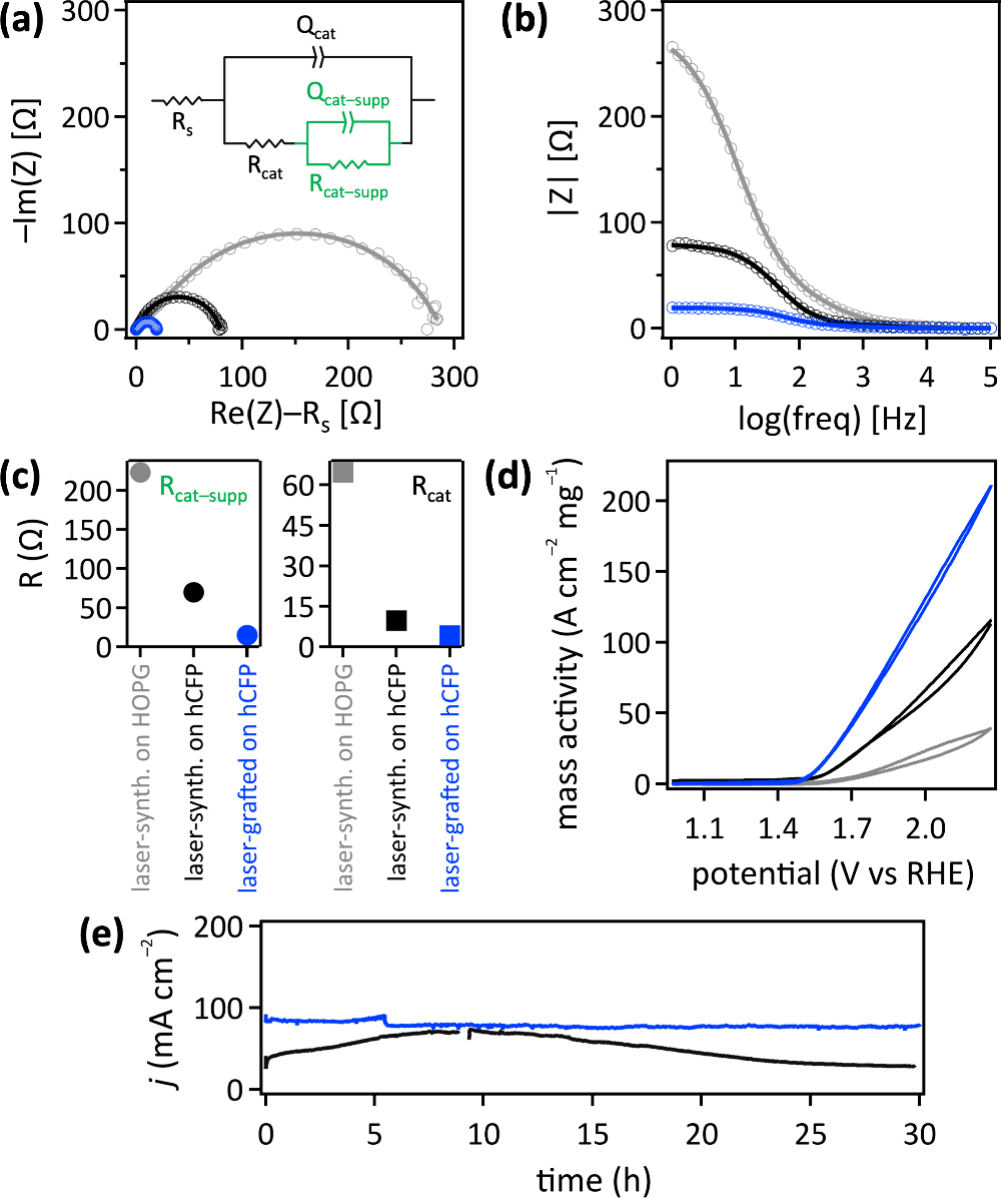
Electrochemistry data of pulsed laser grafted [NiFe]-(OH)_2_ on hydrophilic carbon fiber paper (blue), pulsed laser in liquid synthesized [NiFe]-(OH)_2_ drop cast on hydrophilic carbon fiber paper (black), or pulsed laser in liquid synthesized [NiFe]-(OH)_2_ drop cast on HOPG (gray) in 1.0 M aqueous KOH. (a) Nyquist and (b) Bode plots of PEIS data (open circles) with fits (lines) using the equivalent circuit, shown as an inset in the Nyquist plot, where R_S_ is the solution resistance, R_cat_ the charge transfer resistance through the [NiFe]-(OH)_2_ catalyst material, R_cat–supp_ the charge transfer resistance across the catalyst–support interface, and Q a constant phase element; freq, frequency. (c) Charge transfer resistance values derived from PEIS data; hCFP, hydrophilic carbon fiber paper. (d) Cyclic voltammetry data, collected at a scan rate of 100 mV s^–1^, showing mass activity. (e) Chronoamperometry data at 2.0 V vs RHE, with the current density *j* normalized to the geometric electrode area.

We compared PEIS data of pulsed laser grafted [NiFe]-(OH)_2_ on hydrophilic carbon fiber paper with those of pulsed laser in liquid synthesized [NiFe]-(OH)_2_ drop cast on hydrophilic carbon fiber paper or on flat HOPG. Measurements were conducted in 1.0 M aqueous KOH at 1.7 V vs RHE, in keeping with published data on nickel–iron (oxy)hydroxide catalyzed alkaline water oxidation.^205^ We obtained the smallest semicircle for pulsed laser grafted [NiFe]-(OH)_2_ on hydrophilic carbon fiber paper composites. This indicates that the pulsed laser grafting process enhances the electrical contact between the [NiFe]-(OH)_2_ catalyst and the hydrophilic carbon fiber support, resulting in the lowest impedance. In contrast, using composites consisting of pulsed laser in liquid synthesized [NiFe]-(OH)_2_ nanoparticles deposited via drop casting on hydrophilic carbon fiber paper or HOPG produced higher impedance (Figure 5a–c).

We quantitatively analyzed the PEIS data to derive resistance values, using the equivalent circuit shown in Figure 5a. Our PEIS system can be described as that of hydrated metal (hydr)oxide alkaline water oxidation catalysts, which have extensively been studied.^200−202^ The reported most appropriate equivalent circuit for [NiFe]-(OH)_2_-catalyzed alkaline water oxidation consisted of the uncompensated solution resistance R_S_ in series with an RC loop due to the kinetics of the redox reaction that occurs at the interface between the electrode and the electrolyte, and an RC loop due to the resistivity of the hydroxide film.^201,206^ Here, we deliberately used the [NiFe]-(OH)_2_ catalyst nanoparticles on high surface area carbon fiber paper electrodes to enhance water oxidation performance, creating conditions of fast water oxidation kinetics, manifesting as high mass activity (Figure 5d), so that the charge transfer resistance due to reaction kinetics became negligible, as has been described in the literature.^201^ In contrast to the high surface area anodes that consisted of pulsed laser grafted or pulsed laser synthesized/drop cast [NiFe]-(OH)_2_ on hydrophilic carbon fiber paper, with (real surface area normalized) mass loadings of 0.017 and 0.018 μg cm^–2^, respectively, the [NiFe]-(OH)_2_–HOPG anodes were flat, slowing down water oxidation kinetics, compared to the high surface area carbon fiber paper electrodes. However, for flat electrodes the geometric area is equal to the real surface area, giving rise to a thicker [NiFe]-(OH)_2_ nanoparticle film at the mass loading of 8.8 μg cm^–2^, compared to the [NiFe]-(OH)_2_–carbon fiber paper composites. Therefore, in the case of [NiFe]-(OH)_2_–HOPG anodes, the charge transfer resistance through the layered double hydroxide film outweighed the charge transfer resistance due to the reaction kinetics, thus eliminating the need to include an RC loop associated with the redox reaction kinetics, as reported for scenarios with resistive catalyst films.^201^ Consequently, we did not include an RC loop representing the charge transfer resistance due to reaction kinetics into our circuit model to fit the Nyquist plots of all three anode preparations. This apparent absence of reaction kinetics associated resistance is corroborated by the shape of the Bode plots of all three anode preparations, which each showed two plateaus (Figure 5b), indicative of two resistive elements. A Bode plot graphs the absolute value of the impedance as a function of the logarithm of the frequency, making small impedances identifiable in the presence of large impedances, which may be difficult to observe in a Nyquist plot.^28^ As a result, Nyquist and Bode plots of the same measured impedance data complement each other and enable the derivation of individual resistance values of composite electrodes.^201^

In the reported studies that identified the most appropriate equivalent circuit for [NiFe]-(OH)_2_-catalyzed alkaline water oxidation, nickel–iron hydroxide was used on nickel metal, iron oxide or gold supports,^201,206^ which convert under alkaline water oxidation conditions into the respective surface (oxy)hydroxides or oxides,^5^ so that the resistance at the electrode support–catalyst film interface was designed not to be measurable because of the similarity of the support and catalyst materials under turnover. This leaves the resistivity through the catalyst material as an RC loop in the equivalent circuit (black part of the equivalent circuit in Figure 5a; resistance denoted as R_cat_). However, in the case of carbon being the electrode support material, as used here, the resistivity at the interface between the carbon support and the electrocatalyst material cannot be neglected. Consequently, we included in our equivalent circuit an RC loop that reflects this interfacial catalyst–support resistivity (green part of the equivalent circuit in Figure 5a; resistance denoted as R_cat–supp_). Carbon is the predominant electrode support material for electrocatalysts because of its cost-effectiveness and inertness in many electrochemical reactions.^28,207,208^ Since we observed only two plateaus in the Bode plots, and since the resistance through the [NiFe]-(OH)_2_ material and the resistance across the catalyst–support interface are both due to solid-state resistances and occur simultaneously, we included the RC loop due to R_cat–supp_ in parallel with the RC loop due to R_cat_, instead of in series.^194^ We note that equivalent circuits for catalyst inks of earth-abundant transition metal catalysts on carbon supporting electrodes for alkaline water oxidation^204,209^ are not directly comparable to our scenario because binders alter, and often dominate, the impedance response of integrated electrodes.^210^ In keeping with the literature,^205^ we subtracted the solution resistance, derived from fitting the data using the equivalent circuit shown in Figure 5a from the impedance data shown in the Nyquist and the Bode plots (Figure 5a,b), as to not encumber the data by inherent changes of the solution resistance due to differences in electrode and cell geometry.

We obtained charge transfer resistance values of (15 to 223) ± 3 Ω and (4 to 65) ± 2 Ω (Figure 5c). For [NiFe]-(OH)_2_ on HOPG, we deduced values of 65 ± 2 Ω and 223 ± 3 Ω. A through catalyst resistance of 53 Ω has been reported for nickel–iron layered double hydroxide on a 0.07 cm^2^ glassy carbon supporting electrode,^211^ which is the most closely related system we were able to find in the literature. Given that our HOPG electrode had a geometric area of 0.09 cm^2^, this reported through catalyst resistance of 53 Ω would scale up to 68 Ω for an electrode area of 0.09 cm^2^. Therefore, we infer that the lower resistance values correspond to R_cat_ (Figure 5c). Conversely, the higher resistance values reflect R_cat–supp_, which result from the electrical contact between the [NiFe]-(OH)_2_ catalyst material and the carbon support. Clearly, the pulsed laser grafted [NiFe]-(OH)_2_ on hydrophilic carbon fiber paper composite exhibited the lowest charge transfer resistance across the catalyst–support interface (Figure 5c). This exceptionally low R_cat–supp_ indicates superior electrical contact between the [NiFe]-(OH)_2_ catalyst and the carbon support over composites that consisted of separately laser synthesized [NiFe]-(OH)_2_ nanoparticles that were post synthesis electrostatically attached to the carbon supports. Composites with hydrophilic carbon fiber paper supports displayed lower R_cat–supp_ values than HOPG anodes, presumably because the surface oxygenates at hydrophilic carbon fiber paper^32^ provided stronger electrostatic and electronic interactions with the hydroxide moieties of the [NiFe]-(OH)_2_ nanoparticles than with the HOPG surface. In contrast to graphene, the basal-plane carbon atoms in HOPG are unreactive because of the π-stacking interactions between neighboring graphite sheets, which inhibit basal-plane carbon functionalization by oxygenates.

In addition to exhibiting the lowest charge transfer resistance across the catalyst–support interface, the pulsed laser grafted [NiFe]-(OH)_2_ on hydrophilic carbon fiber paper composite also possessed the lowest charge transfer resistance through the [NiFe]-(OH)_2_ catalyst material (R_cat_, Figure 5c). In general, the conductivity and the thickness of the catalyst layer govern the resistance through the catalyst material.^212^ Unsurprisingly, the [NiFe]-(OH)_2_–HOPG composite exhibited the highest R_cat_ value, owed to the high mass loading of pulsed laser synthesized/drop cast [NiFe]-(OH)_2_ on HOPG of 8.8 μg cm^–2^, normalized to real electrode surface area. In contrast, the hydrophilic carbon fiber paper composites had lower catalyst mass loadings relative to the real surface area due to the high surface area of 468 cm^2^ per geometric cm^2^ of the hydrophilic carbon fiber paper.^8^ Because of the low mass loadings of pulsed laser grafted and pulsed laser synthesized/drop cast [NiFe]-(OH)_2_ on hydrophilic carbon fiber paper of 0.017 and 0.018 μg cm^–2^, respectively, we were unable to measure the conductivities of [NiFe]-(OH)_2_ in the composites. However, in layered double hydroxides, the electrical conductivity increases as the basal plane spacing decreases.^213^ This suggests that the pulsed laser grafted [NiFe]-(OH)_2_ material possessed a higher conductivity than the pulsed laser synthesized [NiFe]-(OH)_2_ nanoparticles, evident from XRD data that showed a smaller basal plane spacing of 6.6 Å for laser grafted [NiFe]-(OH)_2_ compared to 8.0 Å for laser synthesized [NiFe]-(OH)_2_ (Figure 1e and Figure S4). Note that the hydroxide ion conductivity of layered double hydroxides increases with increasing basal plane spacing when the chemical identity of the intercalated anions does not change.^214^ The hydroxide ion conductivity affects the water oxidation kinetics, and we have shown above that our PEIS data did not contain a reaction kinetics associated resistance component. In contrast to the ionic conductivity, the electrical conductivity affects the charge transfer resistance through the material, which is also influenced by the thickness of the catalyst material, dependent on mass loading and nanoparticle aggregation at low specific mass loading. The catalyst mass loading of pulsed laser grafted [NiFe]-(OH)_2_ on hydrophilic carbon fiber paper composites was similar to that of pulsed laser synthesized/drop cast composites. However, the charge transfer resistance through the [NiFe]-(OH)_2_ catalyst material was a factor of 2.3 lower for the pulsed laser grafted composite than for the composite with postsynthesis attached [NiFe]-(OH)_2_ nanoparticles (Figure 5c), indicating that [NiFe]-(OH)_2_ nanoparticle aggregation, in addition to electrical conductivity, governed the through catalyst charge transfer resistance. SEM imaging showed markedly less aggregation of pulsed laser grafted than laser synthesized/drop cast [NiFe]-(OH)_2_ on hydrophilic carbon fiber paper (Figure 1a and Figure S5c). All in all, our data suggest that the pulsed laser grafting process produced [NiFe]-(OH)_2_–carbon fiber paper composites with superior electrical conductivity and more uniform coverage of [NiFe]-(OH)_2_, expected to result in higher electrocatalytic performance.

We found that the water oxidation mass activity of pulsed laser grafted [NiFe]-(OH)_2_ on hydrophilic carbon fiber paper significantly outperformed that of laser synthesized [NiFe]-(OH)_2_ on hydrophilic carbon fiber paper or HOPG (Figure 5d). The alkaline water oxidation activity of integrated [NiFe]-(OH)_2_ on graphitic carbon electrodes was assessed by cyclic voltammetry (CV) in 1.0 M aqueous KOH, in keeping with standard practice.^215^ The CV data of anodes that consisted of pulsed laser grafted [NiFe]-(OH)_2_ on hydrophilic carbon fiber paper additionally showed less hysteresis, owed to the lower impedance and ergo superior electrical contact, than the laser synthesized/drop cast composite materials. Moreover, chronoamperometry data of pulsed laser grafted [NiFe]-(OH)_2_ catalyst on hydrophilic carbon fiber paper composites showed a higher current density and long-term stability than those of an analogous integrated anode that was prepared by laser synthesizing [NiFe]-(OH)_2_ catalyst nanoparticles, followed by drop casting them on hydrophilic carbon fiber paper (Figure 5e). EDX spectra of an anode that consisted of pulsed laser grafted [NiFe]-(OH)_2_ on hydrophilic carbon fiber paper pre and post 30 h water oxidation catalysis show virtually no catalyst loss and no change in the Ni/Fe ratio (Figure S2), corroborating the exceptional stability of the pulsed laser grafted composite.

Overall, our new one-step aqueous methodology of nanosecond pulsed laser grafting of mixed-metal hydroxide catalysts on hydrophilic carbon fiber paper allowed us to fabricate composite anodes that showed decreased impedance, increased water oxidation activity, and enhanced electrocatalytic stability, thus outperforming analogous composites that were prepared by pulsed laser in liquid synthesis, followed by drop casting on the high surface area carbon support. We note that both pulsed laser preparations create nanocatalysts that are surfactant-free, unlike conventional wet-chemistry nanoparticle synthesis methods. Additionally, the pulsed laser grafting process offers superior rapidity and efficiency compared to conventional processes by eliminating sequential nanoparticle synthesis, separation, and postsynthetic attachment steps that are necessary in separate pulsed laser in liquid or conventional synthesis of nanoparticles and subsequent attachment to the support. Furthermore, pulsed laser grafting eliminates the need for binders to enhance the durability of catalyst–support composites under electrocatalytic turnover because catalyst seeding and growth at the graphitic carbon surface enables integrating the transition metal catalyst at the interface of the carbon support, leading to superior adhesion and electrical contact. Leveraging the extensive chemical versatility of reactive pulsed laser in liquid fabrication of nanomaterials,^1^ together with broadly available and well-studied solution redox chemistries,^216−221^ renders pulsed laser grafting universally applicable, with uses in sustainable manufacturing, catalysis, decarbonization technologies, sensing, and biomedical sectors.

## 4 Conclusions

We developed a new methodology for the fabrication of surfactant-free mixed-metal nanocatalyst–support composites with lower impedance, superior electrocatalytic activity and enhanced long-term stability, compared to laser synthesized drop cast analogues. Our innovative pulsed laser grafting process is versatile and broadly applicable. It combines the generation of nanoparticles with their surface attachment into one step, enables utilization of eco-friendly aqueous liquids, and effectively addresses longstanding issues with nanoparticle adhesion and electrical contact in the manufacturing of nanoparticle–support composites. Importantly, pulsed laser grafting is compatible with freestanding, macroscopic solid supports such as carbon fiber paper, surpassing the limitations of powder or particulate supports and distinguishing our approach from previously reported particulate-based systems. Moreover, this method avoids organic solvents and the formation of carbonaceous shells around the nanoparticles, which would hinder catalytic performance. The pulsed laser grafting process also eliminates the need for labor-intensive steps such as separate nanoparticle synthesis, separation, purification, and attachment, thereby addressing common challenges related to preparation time and reproducibility in nanoparticle–support composite fabrication.

We prepared integrated composites of [NiFe]-(OH)_2_ nanocatalysts with a Ni/Fe ratio of 75:25 on hydrophilic carbon fiber paper by pulsed laser grafting, using 1.0 M aqueous (Ni_12_Fe_1_) nitrate solution as grafting liquid and hydrophilic carbon fiber paper as freestanding high surface area support. Hydrophilicity promotes water contact with the carbon fiber paper surface, thereby facilitating interactions with aqueous metal ions and enhancing heat transfer efficiency. We chose 532 nm nanosecond pulses with a fluence of 87 mJ cm^–2^ to minimize carbon surface damage and nonlinear excitation effects in the carbon support during the pulsed laser grafting process. At the selected laser wavelength of 532 nm, both the nickel and the iron nitrate components of the aqueous grafting liquid possess low yet nonnegligible absorbances, evident from optical spectroscopy data. This allows both metal ion compounds to be excited by the laser light while enabling the use of a grafting liquid with a high concentration of metal ions, minimizing laser light attenuation. A high concentration of dissolved metal ions is desired for nanoparticle assembly. We obtained composites with a uniform distribution of [NiFe]-(OH)_2_ on hydrophilic carbon fiber paper, as demonstrated by SEM and EDX data, which detected only C, O, Ni, and Fe. By design, the pulsed laser grafting process avoided unwanted carbon encapsulation of [NiFe]-(OH)_2_, corroborated by XPS data. The pulsed laser grafted [NiFe]-(OH)_2_ nanomaterial was integrated at the interface of the graphitic hydrophilic carbon fiber paper support, as indicated by the prominent Ni–C interactions observed in XPS data, which were absent in laser-synthesized [NiFe]-(OH)_2_ drop-cast on virtually identical hydrophilic carbon fiber paper. This highlights the unique capability of pulsed laser grafting to establish direct chemical interactions between the catalyst and support, enabling a more intimate and more conductive interface than conventional drop-casting methods. The finding of strong Ni–C interactions supports our proposed mechanism for the one-step fabrication of pulsed laser liquid grafted nanoparticle–support composites. The process involves activating the solid substrate surface with the nanosecond laser pulse, inducing metal site seeding from dissolved ions, and promoting the growth of nanoparticles at these seed sites, thereby assembling mixed-metal hydroxide nanoparticles at the carbon support surface.

Pulsed laser grafted composites of [NiFe]-(OH)_2_ on hydrophilic carbon fiber paper displayed enhanced electrocatalytic performance for alkaline water oxidation, compared to analogous composites of laser synthesized [NiFe]-(OH)_2_ nanocatalysts drop cast on hydrophilic carbon fiber paper or HOPG. Analysis of PEIS data of the three composites revealed that our pulsed laser grafting process decreased the charge transfer resistance across the interface between the [NiFe]-(OH)_2_ catalyst nanoparticles and the carbon support. Additionally, the charge transfer resistance through the [NiFe]-(OH)_2_ catalyst material itself was lower. Pulsed laser grafted [NiFe]-(OH)_2_–carbon fiber paper composites showed less [NiFe]-(OH)_2_ nanoparticle aggregation, along with a more uniform coverage of [NiFe]-(OH)_2_, and superior electrical conductivity, leading to increased mass activity for alkaline water oxidation. Further, pulsed laser grafted composites of [NiFe]-(OH)_2_ on hydrophilic carbon fiber paper exhibited exceptional long-term stability, overcoming the ubiquitous adhesion issues of conventional nanoparticle–support composite preparation. All in all, our new pulsed laser grafting methodology to manufacture multimetallic nanoparticle–support composites has the potential to significantly advance electrochemistry and electrocatalysis technologies.

## Supporting Information Available

Pulsed laser grafting setup design and materials characterization data (PDF)
