# Supplementary material for: Fabrication of Surfactant-Free Mixed-Metal Nanocatalyst–Carbon Fiber Paper Composites via Pulsed Laser Grafting
Source: J Phys Chem C Nanomater Interfaces. 2025 Apr 24;129(18):8730–46. doi: 10.1021/acs.jpcc.5c00641 (PMC12067443; doi:10.1021/acs.jpcc.5c00641)
Supplement: Supplementary file 1 — jp5c00641_si_001.pdf [file jp5c00641_si_001.pdf]

Supporting Information

Fabrication of Surfactant-Free Mixed-Metal Nanocatalyst–Carbon Fiber Paper Composites via Pulsed Laser Grafting

Madeleine K. Wilsey,[†] *Teona Taseska,*[‡] *Lydia R. Schultz,*[‡] *Elena Perez,*[‡] *Astrid M. Müller**[,†,‡,§]

[†]Materials Science Program, University of Rochester, Rochester, New York 14627, USA.

[‡]Department of Chemical Engineering, University of Rochester, Rochester, New York 14627, USA.

[§]Department of Chemistry, University of Rochester, Rochester, New York 14627, USA.

Email: Astrid M. Müller - astrid.mueller@rochester.edu
* Corresponding author

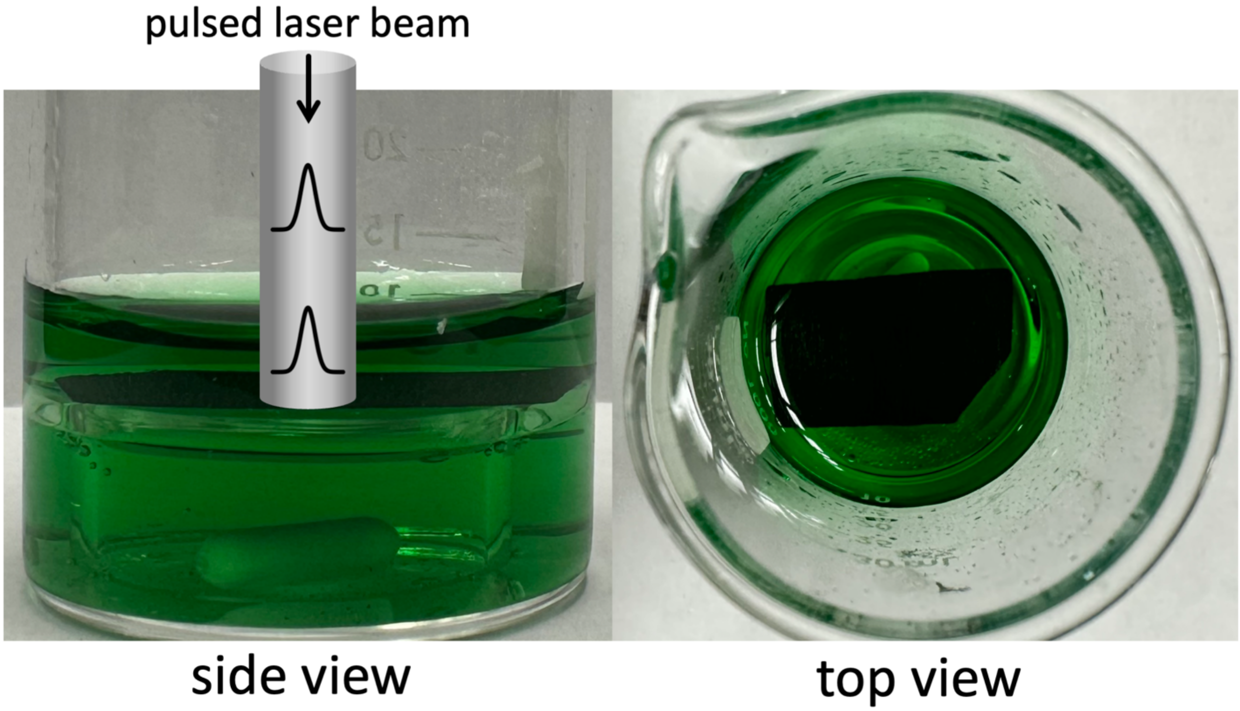

Figure S1. Photographs of the pulsed laser grafting setup for preparing composites of [NiFe]-$(OH)_2$ on hydrophilic carbon fiber paper. On the left, the total reflection of the carbon fiber paper appears as a photographic artifact.

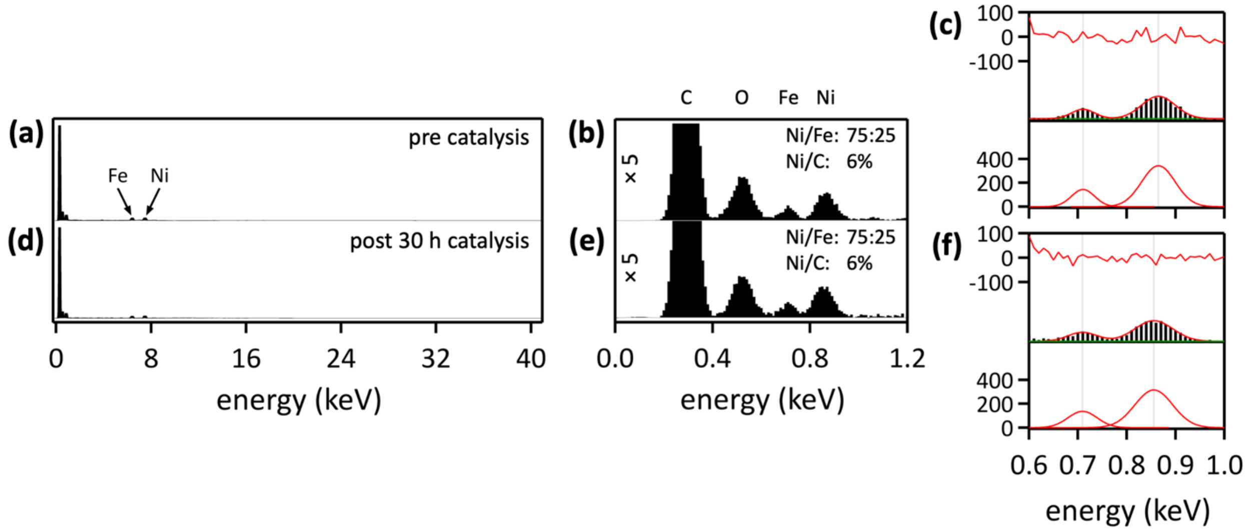

Figure S2. EDX spectra of pulsed laser grafted [NiFe]-$(OH)_2$ on hydrophilic carbon fiber paper composites before and after 30 h of alkaline water oxidation catalysis at 2.0 V vs RHE. Shown are: (a, d) full energy and intensity spectra; (b, e) expanded views of energy and intensity regions; and (c, f) peak fitting of the nickel and iron signals, displaying individual Gaussian components (bottom), experimental data with fitted envelopes (middle), and residuals between the data and fits (top).

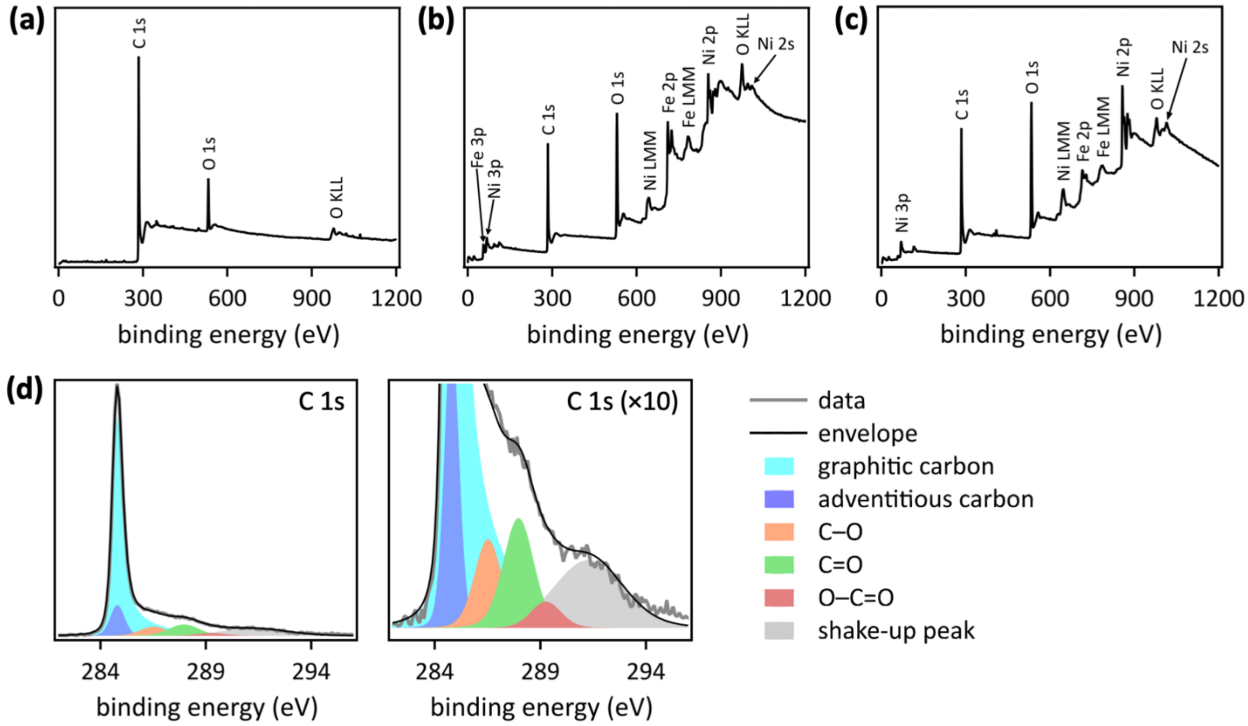

Figure S3. XPS survey spectra for (a) hydrophilic carbon fiber paper, (b) pulsed laser grafted [NiFe]-$(OH)_2$ and (c) laser-synthesized [NiFe]-$(OH)_2$ on hydrophilic carbon fiber paper composites. (d) Core level C 1s XPS data of laser-synthesized [NiFe]-$(OH)_2$ drop cast on hydrophilic carbon fiber paper composite, with full *y*-axis (left) and a *y*-axis magnified by a factor of 10 (right).

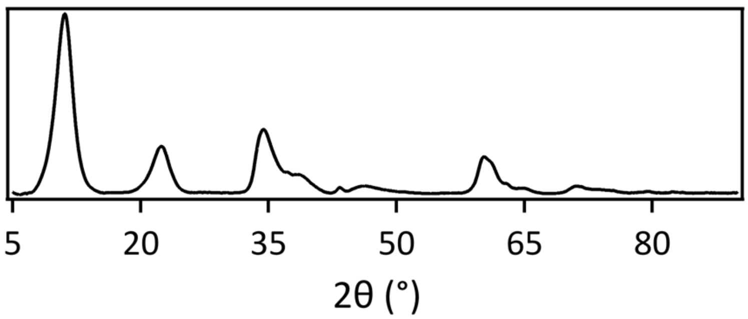

Figure S4. Powder XRD data of laser-synthesized [NiFe]-$(OH)_2$ nanoparticles.

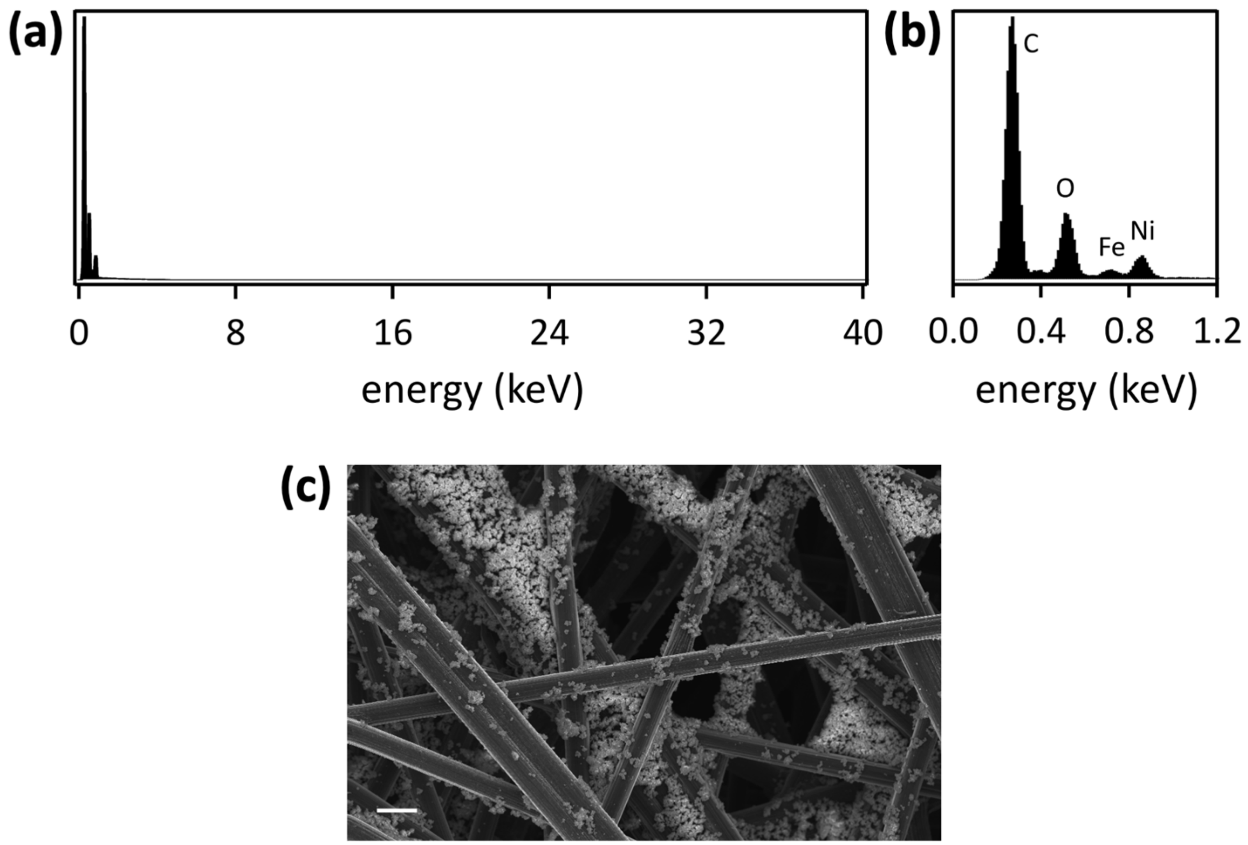

Figure S5. SEM/EDX data of pulsed laser synthesized [NiFe]-$(OH)_2$ drop cast on hydrophilic carbon fiber paper composites, showing EDX spectra over (a) the full energy axis and (b) expanded energy *x*-axis for (c) the corresponding SEM image with a scalebar of 10 μm.

Table S1. Relative contents of C 1s surface oxygenates, the C 1s π–π* shake-up peak, Ni 2p and Fe 2p species with respect to the sum of graphitic and adventitious carbon content, determined from XPS data; sat, satellite.

species	hydrophilic carbon fiber paper		pulsed laser grafted [NiFe]-$(OH)_2$–hydrophilic carbon fiber paper composite	
	central binding energy (eV)	relative content (atom %)	central binding energy (eV)	relative content (atom %)
graphitic carbon	284.7	88.6	284.7	86.30
adventitious carbon	284.8	11.4	284.8	13.70
<u>C</u>–Ni	–	–	285.4	14.08
<u>C</u>–O	286.0	5.4	286.3	3.01
<u>C</u>=O	287.0	4.9	287.0	3.37
O–<u>C</u>=O	289.2	2.3	288.8	6.91
π–π* shake-up peak	291.4	3.7	291.4	5.39
Ni^{2+} $2p_{3/2}$	–	–	854.7	1.35
Ni^{3+} $2p_{3/2}$	–	–	856.1	2.38
sat $2p_{3/2}$	–	–	861.7	3.25
Ni^{2+} $2p_{1/2}$	–	–	872.2	0.54
Ni^{3+} $2p_{1/2}$	–	–	873.7	1.18
sat $2p_{1/2}$	–	–	879.8	1.62
Fe^{2+} $2p_{3/2}$	–	–	711.0	3.60
Fe^{3+} $2p_{3/2}$	–	–	713.0	4.64
sat $2p_{3/2}$	–	–	719.1	1.83
Fe^{2+} $2p_{1/2}$	–	–	724.4	1.78
Fe^{3+} $2p_{1/2}$	–	–	726.5	2.30
sat $2p_{1/2}$	–	–	731.7	2.10
